# HIV-associated depression: a translational framework targeting neuroimmune inflammation and psychosocial stress modulation

**DOI:** 10.3389/fimmu.2025.1645991

**Published:** 2025-09-04

**Authors:** Fei Yu, Yue Zhu, Yiran Fan, Mingqi Chen, Qing Peng, Shenghao Li, Liyuan Hao, Fanghang Ye, Jiajun Xia, Xiaoyu Hu

**Affiliations:** ^1^ School of Clinical Medicine, Chengdu University of Traditional Chinese Medicine, Chengdu, Sichuan, China; ^2^ Department of Infectious Diseases, Zigong First People’s Hospital, Zigong, Sichuan, China; ^3^ Department of Infectious Diseases, Hospital of Chengdu University of Traditional Chinese Medicine, Chengdu, Sichuan, China

**Keywords:** HIV, depression, chronic neuroinflammation, inflammatory cytokines, antiretroviral therapy

## Abstract

People living with HIV (PLWH) are at increased risk for depression, anxiety, and other comorbid psychiatric disorders. HIV-associated depression involves complex neurobiological disturbances, including chronic neuroinflammation. This includes microglial activation, elevated levels of pro-inflammatory cytokines and mediators, and altered brain metabolites. Additionally, there is dysregulation of monoaminergic neurotransmission, particularly impaired serotonergic signaling. Prolonged hyperactivation of the hypothalamic-pituitary-adrenal axis, indicated by abnormally high cortisol levels, is also observed. Together, these pathological processes contribute to persistent brain inflammation and metabolic imbalance. Under prolonged inflammatory conditions, activated microglia release factors such as tumor necrosis factor-alpha. These factors can induce oligodendrocyte apoptosis and demyelination, exacerbating neural injury. Psychosocial stressors—such as stigma, death-related anxiety, and internalized shame—may amplify these pathways through immune-neural crosstalk. Our primary focus, however, is on pharmacological targeting. We propose a three-tiered intervention framework: 1) Targeted neuropharmacological interventions (e.g., SSRIs and anti-inflammatory agents); 2) Optimized ART regimens; 3) Integrated psychosocial support. While further research is needed to establish long-term efficacy and personalized treatment options, this multidimensional approach may reduce the progression of HIV-associated depression and improve clinical outcomes.

## 1 Introduction

HIV/AIDS remains a leading global infectious disease, posing a major public health challenge. According to global HIV/AIDS statistics (1), as of 2023, approximately 39.9 million people worldwide are living with HIV, with approximately 30.7 million receiving antiretroviral therapy (ART) (1). However, funding for AIDS prevention and treatment in low- and middle-income countries reached only $19.8 billion (in 2019 constant dollars) by the end of 2023, reflecting a 7.9% decrease from 2022. Projections indicate that by 2025, these countries will require $29.3 billion (in 2019 constant dollars) for continued AIDS prevention and treatment efforts (1). This funding shortfall represents a critical barrier to eliminating AIDS as a public health threat, while also imposing a substantial economic burden on global healthcare systems.

The widespread use and improved adherence to combination antiretroviral therapy (cART) has significantly increased life expectancy among people living with HIV (PLWH) in both high- and low-income countries, bringing it closer to that of uninfected individuals (2–4). While cART has dramatically reduced HIV transmission, there is currently no cure or vaccine, and HIV infection remains incurable. Consequently, PLWH often experience significant psychological distress, which can stem from various factors, including fear of disease progression—particularly in resource-limited settings—HIV-related stigma, and concerns about long-term health outcomes (5). Comorbid mental health disorders, including depression, anxiety, and other severe psychiatric conditions, are prevalent among PLWH, with depression and anxiety being particularly common (6, 7). Depression has long been recognized as the most prevalent neuropsychiatric disorder associated with HIV infection (8). The global prevalence of depression among people living with HIV (PLWH) is estimated to be 31% (9), significantly higher than the 3.8% observed in the general population (10). This suggests that the risk of depression is approximately eight times greater in PLWH compared to HIV-negative individuals. In the United States, the depression prevalence among PLWH is nearly 30% (11), mirroring the global rate, and anxiety disorders affect 19% of this population (12). By 2030, HIV and depression are projected to become the two leading causes of global disease burden, with these conditions frequently co-occurring (13).

In the PLWH population, depression risk is influenced by gender to a significant extent. Research indicates that women with HIV are at a higher risk of developing moderate to severe depression, while men are more likely to experience moderate depression (14). Data from the Women’s Interagency HIV Study (WIHS), a multicenter cohort of 1,027 HIV-infected women in the United States, reveals a 22.1% prevalence of mood disorders and a lifetime prevalence of major depressive disorder (MDD) at 32.4%, higher than the 22.9% prevalence in the general female population (15). A meta-analysis in China found that the depression prevalence among HIV-positive men ranges from 37.9% to 71.8% (16), with 18.1% of men potentially experiencing severe depression. The severity of depression is notably higher in gay HIV-positive men compared to their heterosexual counterparts, suggesting sexual orientation plays a pivotal role in depression manifestation. Additionally, older male PLWH tend to exhibit more severe depression than younger individuals, highlighting age as a key factor in depression onset. Geographical and economic factors also influence depression prevalence, with higher rates observed in developing and underdeveloped countries. Notably, South America shows the highest prevalence at approximately 44%, while Europe has the lowest, at 22% (9). This disparity is likely linked to the uneven distribution of healthcare resources and research funding, with developed countries offering more medical support and financial resources, exacerbating the depression burden in lower-resource regions (17). In managing long-term treatment and quality of life challenges, PLWH face a heightened risk of mental health issues, particularly anxiety and depression. These psychological conditions are often closely tied to perceived stigma, social withdrawal, insomnia, and guilt (18, 19). Anxiety and depression not only impair cART adherence, increasing the risk of HIV transmission (20), but also frequently involve distressing thoughts of death, suicidal ideation, and self-harm tendencies (2). Further research has demonstrated that PLWH with depression show poorer treatment adherence, directly impacting cART efficacy. One study found that depressed patients were 1.7 times more likely to interrupt their treatment compared to non-depressed individuals, complicating disease management (21).

PLWH are at greater susceptibility for psychiatric disorders, including depression and anxiety; however, the neurobiological mechanisms underlying HIV-associated depression remain poorly understood. This study seeks to elucidate these neurobiological mechanisms and examine the influence of non-clinical factors, offering new perspectives for intervention and treatment. The research is structured around three primary objectives: First, it explores the interaction between psychosocial factors—such as social support, economic stress, and substance abuse—and neurobiological processes, investigating how these factors contribute to the onset and exacerbation of depression. Second, it examines how HIV infection impacts the central nervous system (CNS) through mechanisms such as chronic low-grade neuroinflammation and immune dysregulation, facilitating the development of depression. Third, it evaluates the effectiveness and feasibility of integrated management strategies, combining pharmacological treatments with non-clinical interventions. This study aims to clarify the complex mechanisms of HIV-associated depression and provide a solid theoretical foundation and practical guidance for clinical interventions.

## 2 Multidimensional factors in HIV-associated depression analysis

**Figure 1:**
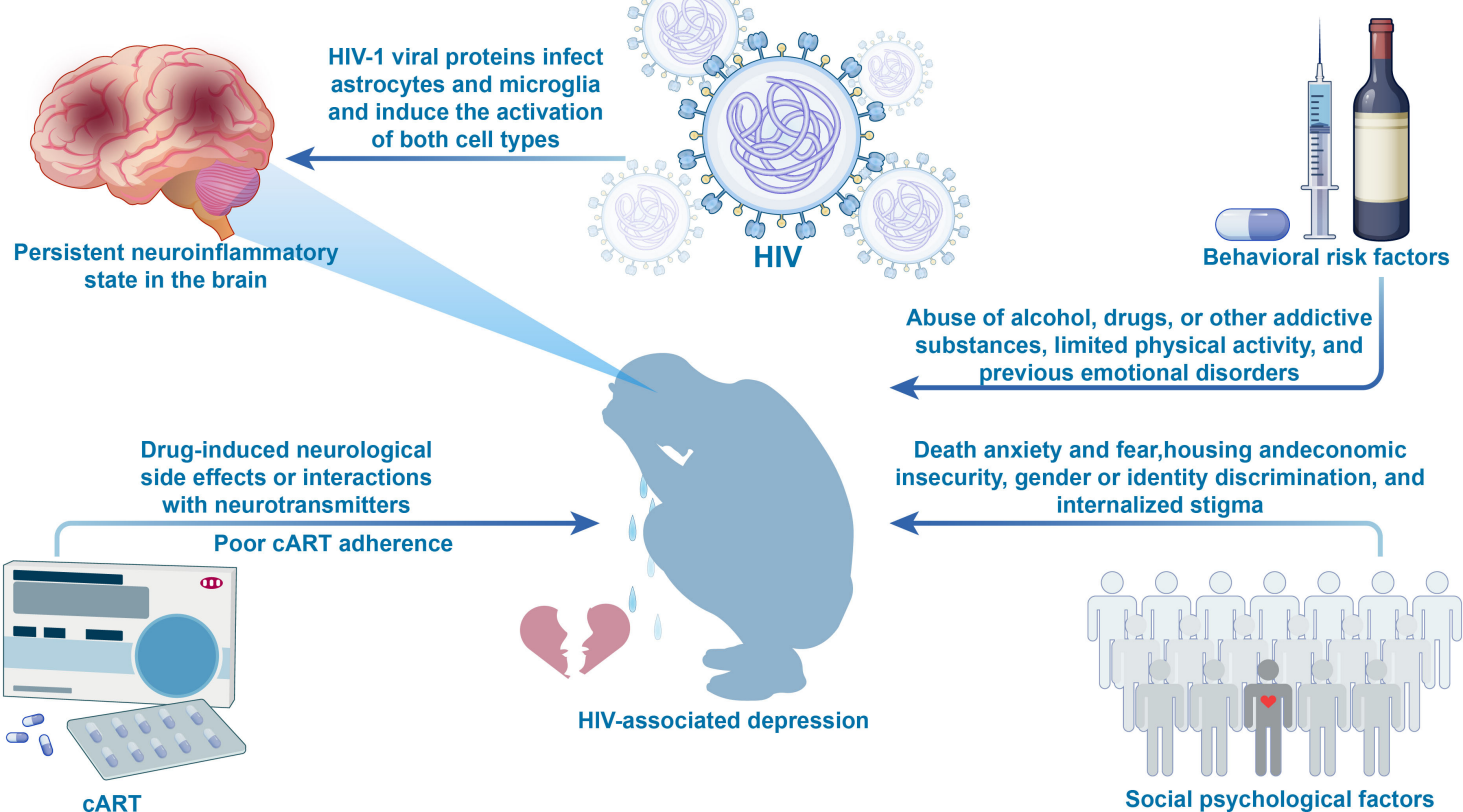
**Figure 1** presents a multidimensional risk factor framework for HIV-associated depression. Biological factors include (1): HIV viral proteins, which activate astrocytes and microglia, leading to persistent brain inflammation (2); Poor adherence to cART, resulting in inadequate viral control and ongoing neuroinflammation (3); cART medications, which may affect neurotransmitter balance, increasing the risk of neuropsychiatric adverse events. Psychological factors include emotional stressors such as death anxiety, negative life events, and internalized stigma. Behavioral factors encompass alcohol and substance abuse, sedentary lifestyle, and a history of mood disorders. Social environmental factors include housing insecurity, economic hardship, limited access to healthcare, violence, and discrimination. The interactions between these factors collectively heighten the risk of depression, offering a comprehensive perspective on its pathogenesis. cART, Combination Antiretroviral Therapy.

The pathogenesis of HIV-associated depression remains incompletely understood; however, research suggests it arises from the interplay of multiple factors, including biological, psychological, and social-environmental influences (**Figure 1**). Various elements contribute to the elevated prevalence of depression among PLWH (22). A study conducted across 11 low- and middle-income countries, involving 2,821 PLWH aged 40 and above, found that unhealthy alcohol consumption and depressive symptoms were most prevalent, with 14%, 9%, and 6% of participants experiencing moderate to severe depression, anxiety, and post-traumatic stress disorder (PTSD), respectively. The comorbidity rate for mental disorders was 11% (23). Behavioral risk factors, including substance abuse, alcohol misuse, and limited physical activity, are also common within this population (15). Alcohol consumption and smoking are the most frequent forms of substance abuse. Systematic reviews and meta-analyses indicate that PLWH globally exhibit higher rates of substance abuse and mental health disorders compared to the general population (24). HIV-related clinical factors, such as poor adherence to ART, are associated with an increased risk of depressive episodes. Additionally, well-established risk factors such as being female, having a history of mood disorders, and a family history of mood disorders are linked to the onset of depression (25). Another study highlighted that PLWH with depression are more likely to engage in substance abuse and risky sexual behaviors, further elevating health risks (26). This finding is supported by another study, which revealed that moderate to high-risk sexual behaviors are more prevalent among PLWH aged ≤30 years, those who are unemployed, and those who have disclosed their HIV status, particularly in individuals with moderate to severe depression (27).

HIV-associated depression is closely associated with socio-psychological risk factors and shows significant temporal and regional variations. In the pre-cART era, death anxiety and PTSD were the primary risk factors (28). However, after the widespread use of cART, structural stressors (such as housing insecurity and disparities in healthcare access) and the internalization of stigma have become increasingly significant (29, 30). This shift manifests differently across regions with varying resources: in low- and middle-income countries, external community discrimination, barriers to treatment accessibility, and internalized stigma are predominant (31), while in high-income countries, factors such as employment discrimination, intimate partner violence, and housing instability are more prominent (32). Notably, social isolation and the lack of a social support system remain important mechanisms across all periods and regions (33, 34). Additionally, PLWH frequently experience substantial stress due to factors such as stigma, discrimination, financial hardship, ART side effects, and unemployment, all of which can lead to depression, anxiety, and suicidal tendencies (35). Intimate partner violence, particularly prevalent among HIV-infected men who have sex with men, is associated with negative behavioral and mental health outcomes (36). Death anxiety and fear are critical factors impacting the quality of life in PLWH. As individuals become aware of the potentially fatal nature of their illness, they may consciously or unconsciously dwell on death, which can trigger anxiety and fear (37). To mitigate these emotions, some PLWH may resort to high-risk behaviors, such as substance or alcohol abuse, as a form of self-soothing. This coping strategy can introduce additional health complications. Furthermore, the presence of death anxiety may distort the interpretation of disease symptoms, increasing the risk of PTSD (38). Death anxiety may also prompt engagement in high-risk sexual behaviors, exacerbating HIV transmission risk (39). Additionally, intense death-related fear and anxiety can lead to social withdrawal, causing PLWH to avoid new social interactions. This not only impacts their social well-being but may create a detrimental cycle of isolation. The intricate relationship between death anxiety, anxiety disorders, and depression is noteworthy, as previous studies have identified high levels of depression as a key determinant of death anxiety (40). In summary, psychological factors such as death anxiety, negative life events, and PTSD—often exacerbated by depression—can drive PLWH to engage in high-risk sexual behaviors, further fueling the spread of HIV. Notably, some individuals living with infectious diseases, including PLWH, may experience improvements in mental health and a reduction in stigma through non-pharmacological interventions, such as psychotherapy and social support (41, 42). These findings underscore the significant role socio-psychological factors play in the onset and progression of HIV-associated depression.

The risk of depression in PLWH is associated not only with medication adherence but also with the neurotoxic effects of certain ART drugs. Studies have shown a negative correlation between depression and ART adherence in PLWH (43, 44). It is important to note that, in addition to the impact of adherence behaviors, the neurotoxic effects of ART medications themselves also represent a significant mechanism. Specifically, ART medications may influence neurotransmitter synthesis, metabolism, or receptor function, thereby indirectly affecting CNS function and elevating the risk of depression. Certain ART drugs, particularly non-nucleoside reverse transcriptase inhibitors (NNRTIs) like Efavirenz (EFV), have been shown to impact the CNS, potentially increasing the risk of depression and other psychiatric disorders by disrupting neurotransmitter equilibrium, interfering with metabolic pathways, and altering neurophysiological processes (45, 46). In EFV users, the incidence of neuropsychiatric adverse drug reactions (ADRs) approaches 50%, with common side effects including dizziness, sleep disturbances, vivid dreams, mood disorders, severe depression, and anxiety disorders (47). Research suggests EFV may induce neurotoxicity by impairing mitochondrial function and energy metabolism in the brain, processes linked to cognitive decline and depression (48). However, further clinical and experimental validation is required to confirm these effects. ART medications may also induce neurotoxicity through mechanisms such as dendritic spine damage, mitochondrial dysfunction, oxidative stress, endoplasmic reticulum stress, and alterations in neuronal growth and synaptogenesis *in vitro*. Collectively, these mechanisms contribute to an elevated risk of depression and other ADRs (49, 50). Compared to monotherapy, ART regimens may lead to a higher incidence of CNS-related adverse reactions and discontinuation. For example, the risk of discontinuation due to CNS adverse effects is 1.92 times higher when dolutegravir (DTG) is combined with abacavir (ABC) compared to DTG monotherapy (51).

HIV activates the NLRP3 inflammasome in microglial cells upon infection, leading to the activation of both microglial cells and astrocytes, which fosters chronic inflammation in the brain and heightens the risk of depression. HIV-1 viral proteins trigger the NLRP3 inflammasome in brain microglia *via* the TLR2-NF-κB signaling pathway, initiating microglial activation (52–54), intensifying the inflammatory response, and contributing to neuronal dysfunction or neurotoxicity (55, 56). Furthermore, research has linked NLRP3 inflammasome activation with HIV-associated neuropathological changes, suggesting that HIV infection can induce harmful neuroinflammatory responses in the CNS (57). It is important to note that the aforementioned HIV-induced neuroinflammatory mechanisms are primarily observed during the untreated viremia phase. However, in the context of cART treatment, residual inflammation may persist even after viral suppression is achieved. Clinically, an early study measuring plasma inflammatory cytokines in 23 PLWH revealed that, compared to non-depressed controls, patients with depression exhibited elevated levels of IL-15, IP-10, IL-12, and granulocyte colony-stimulating factor (G-CSF) (58). Subsequent research, including a multicenter cohort study involving 1,727 participants, demonstrated a strong interaction between chronic depressive symptoms and elevated inflammation levels (59–61). A large meta-analysis supports the bidirectional relationship between inflammation and depression (62). Notably, C-reactive protein (CRP), a peripheral inflammation marker, has been extensively studied for its association with both the incidence and severity of depression (63). Even after adjusting for clinical and psychosocial factors, CRP levels remain significantly correlated with depression (64). Further studies suggest that elevated CRP may be a consequence of depression, whereas elevated IL-6 levels are more likely to be a risk factor, implicating the IL-6/IL-6R signaling pathway in depression pathophysiology (65, 66). It should be emphasized that the detection of CRP and IL-6 in these studies is aimed at assessing the chronic neuroinflammatory burden in PLWH, and these nonspecific pro-inflammatory cytokines and other inflammatory markers may not be suitable to be considered as diagnostic indicators of depression.

Moreover, HIV infection has been shown to induce alterations in brain metabolites, which reflect ongoing CNS inflammation and suggest continuous neural damage (67, 68). Neurofilament light chain protein (NfL) has emerged as a novel biomarker for neuronal injury and neuroinflammation. Studies indicate that NfL concentrations are significantly elevated in the blood and cerebrospinal fluid (CSF) of PLWH, with levels correlating to the severity of depressive symptoms (69–71). As aging progresses, the inflammatory state in the brain intensifies, and microglial dysfunction and neuronal damage worsen (72). These findings suggest that HIV infection not only triggers neuroimmune activation but may also exacerbate age-related neural damage. In summary, the long-term effects of HIV infection on the nervous system may involve a complex interplay between chronic neuroinflammatory responses and age-related neuronal damage, ultimately impacting brain health.

## 3 Neuropathological changes in HIV-associated depression

### 3.1 Inflammatory and metabolic dysregulation in HIV-associated depression

**Figure 2:**
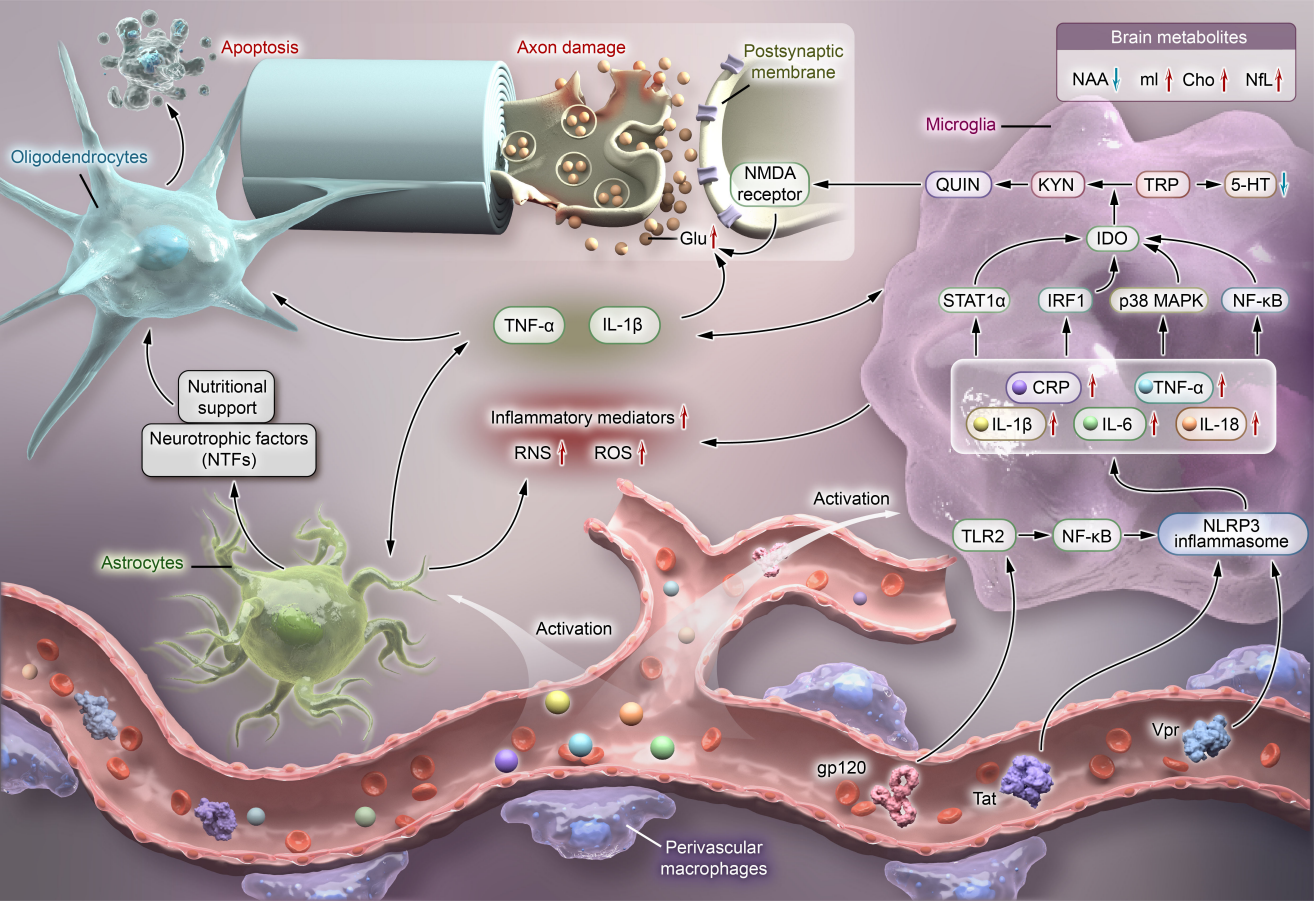
**Figure 2** illustrates the complex interaction between inflammation and metabolic dysregulation in HIV-associated depression, emphasizing the central role of microglial activation in driving both inflammatory and metabolic disturbances. HIV viral proteins activate NLRP3 inflammasomes in microglial cells, which triggers the activation of both microglia and astrocytes. This activation promotes the release of pro-inflammatory factors (such as IL-1β, IL-6, CRP, and TNF-α) and inflammatory mediators, while also increasing the production of ROS and RNS. IL-1β and TNF-α modulate the expression of glutamate receptors and enhance Glu release, leading to excessive accumulation of Glu in the synaptic cleft. This, in turn, stimulates glial cells to secrete additional pro-inflammatory cytokines, amplifying the inflammatory signals within the CNS. Moreover, these inflammatory cytokines activate IDO *via* signaling pathways such as STAT1α, IRF-1, p38 MAPK, and NF-κB, resulting in a metabolic shift from TRP to KYN. This shift not only decreases 5-HT synthesis but also promotes the overproduction of QUIN. By activating NMDA receptors on the postsynaptic membrane, QUIN promotes glutamate release, resulting in the excessive accumulation of neurotransmitters and subsequent neurotoxicity. Concurrently, chronic inflammation and excessive glial cell activation release TNF-α, further exacerbating neuroinflammation by inducing oligodendrocyte apoptosis and demyelination, thereby damaging the nervous system. Additionally, alterations in brain metabolites, such as reduced NAA and elevated NfL, mI, and Cho, indicate ongoing neuroinflammation and potential axonal damage. Together, these mechanisms of inflammation and metabolic dysregulation sustain chronic inflammation within the CNS, increasing the susceptibility to depression in HIV-infected individuals. CNS, central nervous system; Glu, glutamate; ROS, reactive oxygen species; RNS, reactive nitrogen species; IDO, indoleamine 2,3-dioxygenase; TRP, tryptophan; KYN, kynurenine; QUIN, quinolinic acid; 5-HT, serotonin; NMDA, N-methyl-D-aspartate; mI, myo-inositol; NAA, N-acetylaspartate; Cho, choline; NfL, neurofilament light chain; NTFs, neurotrophic factors; CRP, C-reactive protein; TNFα, tumor necrosis factor-α; IL-1β, interleukin-1β; IL-6, interleukin-6; IL-18, interleukin-18; gp120, human immunodeficiency virus envelope glycoprotein 120; Tat, trans-activator of transcription; Vpr, viral protein R.

Neuroinflammatory responses likely play a central role in the neuropathology of HIV-associated depression (**Figure 2**). The complex interplay between immune suppression and activation, elevated peripheral pro-inflammatory cytokines and other inflammatory markers, and cytokine involvement in neurophysiology suggests that inflammation is a key pathological mechanism underlying depression (73–76). Research has shown that low-grade inflammation and excessive cytokine secretion in peripheral blood are closely linked to HIV-associated depression (77). The “cytokine hypothesis of depression” proposes that inflammatory cytokines act as neuromodulators, influencing behavior, neuroendocrine function, and neurochemical characteristics associated with depression (78, 79). Clinical studies consistently report elevated levels of pro-inflammatory cytokines and other inflammatory markers, such as IL-1β, IL-6, CRP, and TNF-α, in individuals with depressive symptoms (80–82). Additionally, exogenous inflammation induction can lead to the development of core depressive features (83).

In HIV-infected individuals, the brain is a primary target for the virus, predominantly affecting perivascular macrophages, microglia, and astrocytes (84, 85). For instance, HIV-1 envelope glycoprotein 120 (gp120) activates microglial cells *via* the TLR2-NF-κB signaling pathway, triggering NLRP3 inflammasome activation and pro-inflammatory cytokine secretion (53, 55). Other viral proteins, such as HIV-Tat and Vpr, further activate microglial cells through multiple signaling pathways, exacerbating inflammatory responses and neurotoxicity, which leads to neuronal dysfunction and severe CNS damage (52, 54, 56). The synergistic effects of these viral proteins highlight the complexity of HIV-induced CNS damage, involving intricate inflammatory and neurotoxic pathways.

In an inflammatory state, microglia and astrocytes further amplify the secretion of pro-inflammatory cytokines such as IL-1β, IL-6, IL-18, and TNF-α (86). IL-1β and TNF-α regulate the expression of glutamate (Glu) receptors and enhance Glu release, causing excessive accumulation of Glu in the synaptic cleft. This not only promotes further cytokine release from glial cells but also amplifies inflammatory signaling within the CNS. Additionally, these cytokines trigger the production of reactive oxygen species (ROS) and reactive nitrogen species (RNS), directly damaging neurons and inducing oligodendrocyte apoptosis (87, 88). Chronic inflammation and TNF-α release by hyperactivated glial cells exacerbate neuroimmune activation by promoting oligodendrocyte apoptosis and demyelination, thereby contributing to progressive CNS damage (89, 90).

Metabolic dysregulation plays a critical role in HIV-associated depression. Depression itself is closely linked to impaired serotonin (5-HT) function, and HIV infection may exacerbate this through several pathways, including inflammation and stress. One such pathway is the metabolic shift from tryptophan (TRP) to kynurenine (KYN), which has been strongly associated with depression development (91, 92). As a precursor to 5-HT, the diversion of TRP towards the KYN pathway reduces 5-HT synthesis while promoting the overproduction of the neurotoxic metabolite quinolinic acid (QUIN), further amplifying neuroinflammation and neurotoxicity (93, 94). Inflammatory cytokines activate indoleamine 2,3-dioxygenase (IDO) *via* signaling pathways such as STAT1α, IRF-1, p38 MAPK, and NF-κB, driving this metabolic shift and contributing to neurotransmitter dysfunction linked to depression (95–97).

Although cART enables most PLWH to achieve viral suppression, some patients still exhibit residual neuronal dysfunction and abnormalities in neuroinflammatory biomarkers. In untreated HIV-1-infected individuals, CSF levels of sTREM2 not only increase with disease progression (as indicated by CD4^+^ T cell depletion) but are also closely associated with NfL levels (98). While PLWH who achieve viral suppression show an overall decrease in sTREM2 levels, neurodamage biomarkers, such as NfL, can still be detected, suggesting the presence of irreversible damage or low-level persistent inflammation (69–71). These changes are not only closely linked to the severity of depression but may also exacerbate HIV-related cognitive and emotional impairments.

HIV infection and inflammatory states also disrupt the balance of brain metabolites. Elevated levels of neurometabolites, such as myo-inositol (mI), have been linked to both the incidence and severity of depression (99). While cART partially mitigates HIV-induced neuroinflammation and metabolite changes (67, 100), neuronal dysfunction (evidenced by reduced N-acetylaspartate [NAA]) and neuroinflammation (indicated by increased mI and choline [Cho]) persist in chronically infected individuals, often accompanied by axonal damage (68, 100).

In summary, HIV disrupts the balance between neuroprotection and neurotoxicity within the CNS through neurotransmitter dysfunction, metabolic dysregulation, and the sustained elevation of pro-inflammatory cytokines. The excessive activation of microglia and astrocytes, driven by inflammation, amplifies these processes, ultimately contributing to the onset of depression.

### 3.2 Microglial activation in the metabolic and inflammatory mechanisms of HIV-associated depression

Microglial activation linked to depression primarily occurs in the prefrontal cortex, insula, and anterior cingulate cortex, with neuronal and synaptic damage resulting from this activation serving as a key trigger for depressive-like behaviors. This activation is strongly associated with neuropsychiatric disorders, including depression and anxiety (101, 102). The process typically involves the secretion of pro-inflammatory cytokines, which initiate both peripheral and central inflammation. In HIV infection, glucose metabolism is significantly upregulated, as evidenced by increased glucose transport and metabolic flux (103, 104). This metabolic alteration is closely tied to heightened production of pro-inflammatory cytokines, such as CRP, TNF-α, and IL-6 (105, 106). These cytokines can cross the blood-brain barrier and affect key brain regions like the amygdala, hippocampus, hypothalamus, and cortex, influencing pathophysiological processes associated with depression, including neurotransmitter metabolism, neuroendocrine function, and neuroplasticity (107). These disruptions further impair mood and cognitive function, providing a critical pathological basis for depression.

### 3.3 Metabolic dysregulation and inflammation in ART-treated PLWH

Although the neurotoxicity of the latest generation of ART has been significantly reduced, treated individuals still exhibit metabolic abnormalities. These metabolic shifts may perpetuate inflammation and immune dysfunction, thereby increasing the risk of depression (108). Notably, while ART partially normalizes certain depression-related inflammatory cytokines in HIV-infected individuals, others, such as IL-6 and CRP, remain elevated (109, 110). Further research has highlighted significant changes in CNS metabolites in ART-treated PLWH through CSF metabolomics, including alterations in neurotransmitters (e.g., Glu and NAA), microglial activation markers (e.g., mI), and ketone bodies (e.g., β-hydroxybutyrate and 1,2-propanediol) (111). These alterations suggest ongoing inflammation, microglial activation, and glutamate-mediated neurotoxicity (111). Despite improvements in viral control and immune function, studies indicate that HIV-associated cognitive impairments and brain inflammatory changes persist in PLWH (67). These findings suggest that ART does not fully resolve CNS pathological changes induced by HIV infection, with underlying mechanisms likely related to persistent inflammation and metabolic dysregulation.

### 3.4 HPA axis dysregulation in HIV-associated depression

The hypothalamic-pituitary-adrenal (HPA) axis plays a critical role in regulating the stress response through the coordinated interaction of the hypothalamus, pituitary gland, and adrenal glands. Under stress, the hypothalamus releases corticotropin-releasing hormone (CRH), which stimulates the pituitary to secrete adrenocorticotropic hormone (ACTH), ultimately prompting the adrenal glands to release cortisol. Cortisol, in turn, modulates the secretion of CRH and ACTH through a negative feedback mechanism to maintain homeostasis. In PLWH, prolonged activation of the HPA axis can occur due to various stressors, such as emotional stress (e.g., death anxiety, stigma, and discrimination) and economic pressure. These factors exacerbate psychological burden and disrupt neuroendocrine function through abnormal cortisol levels (112). Research has shown that PLWH are particularly susceptible to HPA axis dysfunction and fatigue, heightening the risk of HIV-associated mood disorders (113, 114). For instance, the HIV-Tat protein, in response to stress, can induce neurodegeneration and impair HPA axis function, leading to excessive activation and elevated cortisol levels—key mechanisms driving HIV-related neuroendocrine dysfunction and mood symptoms (115, 116). However, studies on HPA axis alterations in PLWH remain scarce, warranting further investigation into the underlying mechanisms and their impacts.

## 4 Potential clinical interventions and challenges in HIV-associated depression

**Figure 3:**
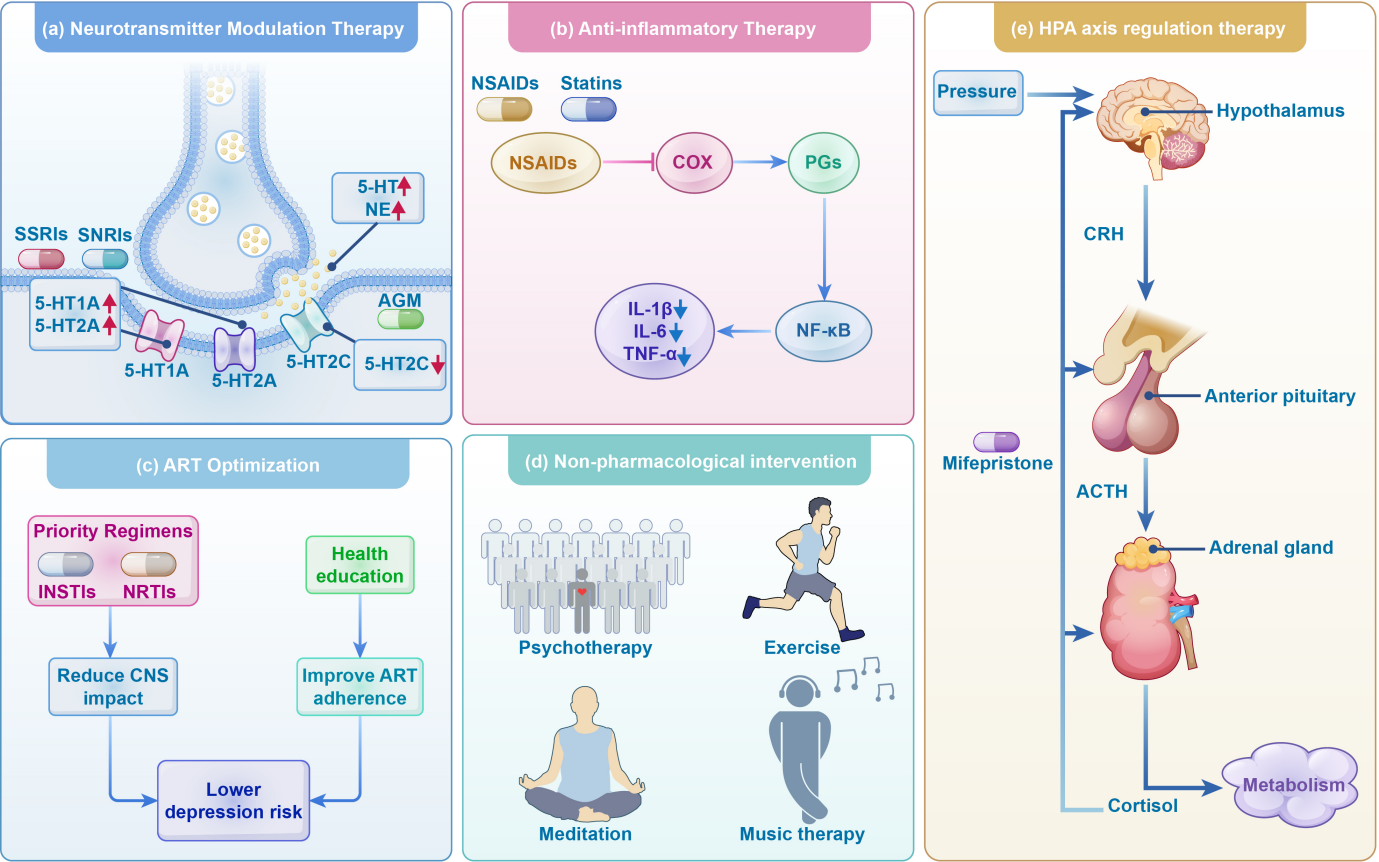
Treatment strategies for depression in HIV infection. This figure delineates treatment approaches for depression in individuals with HIV, encompassing antidepressant therapies such as neurotransmitter modulation, anti-inflammatory treatments, ART optimization, and HPA axis regulation, alongside the use of non-pharmacological interventions as adjunctive therapies. SSRIs, Selective serotonin reuptake inhibitors; SNRIs, Selective norepinephrine-serotonin reuptake inhibitors; AGM, agomelatine; 5-HT, serotonin; 5-HT1A, 5-Hydroxytryptamine 1A receptors; 5-HT2A, 5-Hydroxytryptamine 2A receptor; 5-HT2C, 5-hydroxytryptamine 2C receptors; NSAIDs, Non-steroidal anti-inflammatory drugs; COX, cyclooxygenase; PGs, prostaglandin; CRH,corticotropin-releasing hormone; ACTH, adrenocorticotropic hormone; INSTIs, integrase strand transfer inhibitors; NRTIs, nucleoside reverse transcriptase inhibitors. ART, antiretroviral therapy.

In the management of depression in the context of HIV infection, three primary interventions are typically employed (1): Antidepressant medications and adjunctive treatments with antidepressant effects (2); Non-pharmacological approaches, including exercise therapy and psychosocial interventions, as outlined in **Figure 3**; and (3) Optimization of ART to mitigate the risk of HIV-associated psychiatric disorders. Each intervention presents distinct advantages and limitations, necessitating a tailored approach based on the unique characteristics of the individual PLWH. The specific implementation and suitability of these strategies will be explored further in the subsequent sections.

### 4.1 Neurotransmitter modulation in depression therapy

Selective serotonin reuptake inhibitors (SSRIs) represent an important treatment strategy for managing HIV-associated depression by improving mood symptoms while also modulating neuroinflammation and neurodegeneration. SSRIs exert their therapeutic effects primarily by inhibiting the reuptake of 5-HT into presynaptic neurons, which results in increased 5-HT concentrations in the synaptic cleft. This boost in serotonin levels enhances binding to postsynaptic receptors such as 5-HT1A and 5-HT2A, thereby facilitating improved signal transmission (117, 118). With prolonged use, the enhanced 5-HT signaling helps regulate neural network function, leading to clinical improvements in mood disorders like depression and anxiety. In addition to their mood-regulating effects, SSRIs may also have anti-inflammatory and neuroprotective properties, making them a promising adjunctive therapy for depression in PLWH through their anti-inflammatory and neuroprotective effects (119). Studies have shown that SSRIs can reduce CNS inflammation and neuronal damage. For example, escitalopram has demonstrated the ability to repair HIV-1-mediated neuronal damage and improve neurological function (120). SSRIs may also downregulate the expression of CD4 receptors and chemokine receptors, reducing immune cell susceptibility to HIV infection, further supporting their potential as adjunctive therapies (121). However, despite the benefits, long-term SSRI use can lead to alterations in 5-HT receptor sensitivity, which may trigger side effects such as sexual dysfunction, mood fluctuations, weight changes, and even drug resistance (122). These potential adverse effects require careful monitoring and consideration in clinical practice.

Selective norepinephrine-serotonin reuptake inhibitors (SNRIs) enhance neurotransmitter concentrations in the synaptic cleft by inhibiting the reuptake of 5-HT and norepinephrine (NE), thus improving mood and alleviating depressive symptoms (123). Compared to SSRIs, SNRIs may offer superior efficacy in treating depression and anxiety, particularly in cases linked to NE deficiency. Mirtazapine, for example, due to its sedative and appetite-stimulating properties, coupled with relatively mild gastrointestinal side effects, may be preferred over SSRIs in certain patient populations (124). Additionally, SNRIs exert beneficial effects by reducing neuroinflammation through inhibition of pro-inflammatory cytokine release and modulation of inflammatory pathways, demonstrating significant efficacy in patients with major depressive disorder (125–127). However, prolonged SNRI use in HIV-associated depression treatment may lead to adverse reactions, such as drug interactions, liver toxicity, and withdrawal symptoms. Venlafaxine, for instance, has been associated with increased risk of sexual dysfunction, anorexia, nausea, and insomnia (128). Withdrawal reactions, including dizziness, anxiety, and emotional instability, have also been reported with SSRIs and SNRIs (e.g., venlafaxine and paroxetine) (129, 130), underscoring the importance of a gradual tapering process to prevent abrupt discontinuation. In conclusion, while SNRIs present distinct advantages in the treatment of depression, their potential adverse effects and drug interactions must be meticulously managed to ensure clinical safety and efficacy.

The concomitant use of SSRIs or SNRIs with ART drugs may lead to altered plasma concentrations and clinical adverse reactions. Most antidepressants are substrates of CYP2D6, CYP2B6, or CYP3A4, and interactions between certain ART drugs and antidepressants are primarily mediated through the CYP450 enzyme system. Protease inhibitors (PIs) and NNRTIs can significantly alter antidepressant concentrations by inhibiting or inducing these isoenzymes. Studies have shown that high-dose ritonavir can cause significant changes in the area under the curve (AUC) and maximum plasma concentration of desipramine and trazodone (131). Additionally, the combination of fosamprenavir-ritonavir may reduce the AUC of paroxetine by up to 55% (132). Furthermore, case reports suggest that co-administration of darunavir/ritonavir with duloxetine and aripiprazole may increase duloxetine exposure due to slowed metabolism and elevate the risk of aripiprazole toxicity (133). Therefore, for HIV-infected individuals with impaired liver function, the following measures are recommended: 1) avoid the concomitant use of potent CYP inhibitors (e.g., high-dose ritonavir combined with fluoxetine may trigger serotonin syndrome (134)); 2) regularly monitor liver function and drug concentrations; 3) closely monitor patients for drug-related adverse events when initiating antidepressant therapy and adjust the antiretroviral treatment regimen dosage based on individual responses.

Agomelatine (AGM) is an innovative antidepressant with a distinct mechanism of action, involving the activation of melatonin receptors (MT1/MT2) and inhibition of 5-hydroxytryptamine 2C receptors (5-HT2C). This dual action effectively regulates circadian rhythms while enhancing mood and sleep functions, making AGM particularly suitable for patients with depression accompanied by sleep disturbances or circadian rhythm disruptions (135). Additionally, AGM promotes neuroplasticity by activating melatonin receptors and upregulating brain-derived neurotrophic factor (BDNF) expression, demonstrating notable therapeutic effects in patients with depression linked to sleep disorders and neurofunctional impairments (136). Research indicates that AGM is generally well-tolerated, although it may lead to elevated liver enzyme levels; therefore, regular liver function monitoring is advised during the early stages of treatment (137). However, the use of AGM presents certain limitations (138): first, its efficacy depends on the extent of circadian rhythm disruption and the patient’s specific condition, with more pronounced effects seen in those with significant circadian disturbances; second, while its sleep-enhancing effects are rapid, antidepressant effects typically take several weeks to manifest, similar to other antidepressants.

### 4.2 Anti-inflammatory-based treatment strategies for depression

Recent studies have highlighted that depression is not only a mental disorder linked to neurotransmitter imbalances but also closely associated with chronic low-grade inflammation. This insight suggests that anti-inflammatory agents could serve as potential adjunctive treatments for depression. Previous research has shown that various anti-inflammatory drugs, including non-steroidal anti-inflammatory drugs (NSAIDs), statins, cytokine inhibitors, glucocorticoids, and minocycline, demonstrate therapeutic potential in alleviating depression and its associated symptoms (139). As noted, depression is strongly connected to chronic inflammation, and NSAIDs, by inhibiting cyclooxygenase (COX) activity and reducing prostaglandin (PG) synthesis, indirectly regulate the NF-κB signaling pathway, thus decreasing the release of pro-inflammatory cytokines like interleukins and TNF-α, which may mitigate the inflammatory responses involved in depression (140). While earlier studies suggested that NSAIDs might have minimal effects on depressive symptoms (141), more recent research indicates that anti-inflammatory treatments, including NSAIDs, can improve depressive symptoms in patients with MDD (139, 142). However, further studies are needed to identify specific patient subgroups who may benefit most from these treatments. Bai et al. also emphasized that NSAIDs, alongside other anti-inflammatory agents such as Omega-3 fatty acids, statins, and minocycline, exhibit antidepressant effects in both monotherapy and adjunctive therapy for MDD, with favorable safety profiles (143). Furthermore, TNF antagonists have shown promise as adjunctive therapies to enhance antidepressant efficacy in treatment-resistant depression (144), and patients with MDD may also benefit from corticosteroids and NSAIDs (145). However, the long-term use of NSAIDs is associated with gastrointestinal side effects (such as ulcers and bleeding), an increased risk of cardiovascular events, and renal dysfunction. Consequently, NSAIDs are typically regarded as adjunctive treatments rather than primary therapeutic options for depression.

Statins may play a potential role in the adjunctive treatment of depression through several mechanisms, including anti-inflammatory effects, enhancement of cerebrovascular function, and neurovascular protection (146, 147). Recent studies further support their application in the prevention and management of depression. A cohort study utilizing the UK Biobank, which involved over 360,000 participants, found that regular statin use was associated with a reduced risk of depression (HR = 0.87, 95% CI: 0.81 – 0.94), with consistent results in sensitivity analysis (148). Similarly, a Swedish registry study encompassing more than 1.14 million individuals corroborated this finding, revealing that statin use was linked to a reduced risk of depressive disorders (HR = 0.91, 95% CI: 0.87 – 0.94), a result that remained robust even after adjusting for antidepressant use (HR = 0.91, 95% CI: 0.88 – 0.94) (149). Moreover, a comparison between fluoxetine monotherapy and combination therapy with high-dose atorvastatin (80 mg/day) demonstrated a reduction in pro-inflammatory cytokines and other pro-inflammatory cytokines and other inflammatory markers such as NLRP-3 and IL-6, as well as improved Hamilton Depression Rating Scale scores (150). These findings suggest that atorvastatin may be a promising adjunctive therapy for patients with MDD by modulating the AMPK/NLRP3 and IL-6/STAT-3 signaling pathways (150). Although these studies provide compelling evidence for the potential of statins in depression prevention and intervention, the pharmacokinetic properties and drug interaction risks of statins require careful consideration. Statins such as simvastatin, lovastatin, and atorvastatin are primarily metabolized *via* CYP3A4. When co-administered with strong CYP3A4 inhibitors (e.g., ritonavir, saquinavir), these statins may exhibit significantly increased blood concentrations, raising the risk of severe adverse effects such as rhabdomyolysis and liver damage (151). In contrast, statins with lower drug interaction risks, such as pravastatin and rosuvastatin, are cleared *via* non-hepatic enzymes and may be more suitable for special populations, including PLWH. Therefore, for patients requiring concomitant use of CYP3A4 inhibitors, pravastatin or rosuvastatin is preferred, with dose adjustments and close monitoring of muscle and liver function markers to minimize the risk of adverse effects.

### 4.3 HPA axis-based treatment strategies for depression

As discussed in Section 3.4, PLWH who also suffer from depression often exhibit excessive activation of the HPA axis, leading to abnormally elevated cortisol levels. Consequently, interventions targeting the HPA axis are of considerable clinical and theoretical significance for treating depression, offering a scientific basis for optimizing antidepressant strategies and advancing personalized medicine. Cortisol, a central hormone in the stress response, when chronically elevated, can exert neurotoxic effects on brain regions such as the hippocampus and prefrontal cortex, which are essential for memory and emotional regulation. This prolonged exposure to elevated cortisol can lead to neuronal atrophy and functional impairment (152, 153). Mifepristone, a glucocorticoid receptor (GR) antagonist, exerts its therapeutic effects by blocking GR, thereby rectifying HPA axis overactivation, restoring negative feedback regulation, and lowering cortisol levels. This mechanism helps to reduce the stress-induced hyperactivity of the HPA axis, alleviate cortisol-induced neurotoxicity in the hippocampus and prefrontal cortex, and potentially provide neuroprotective effects in these regions (154, 155). In summary, mifepristone has shown potential efficacy in certain depression subtypes, such as treatment-resistant depression or cortisol-related depression. However, due to its antagonistic effects on progesterone receptors, its use in female patients, particularly those of reproductive age, warrants caution. While preliminary studies have shown promising results, its efficacy and safety must be confirmed through large-scale, long-term clinical trials to better define its practical application in depression treatment.

### 4.4 Application of non-pharmacological interventions in adjunctive treatment of depression

While pharmacological treatment remains the cornerstone of depression management in PLWH, concerns regarding its potential adverse effects—such as physical discomfort, sexual dysfunction, substance abuse, and overdose-related fatalities—along with the interactions between antidepressants and antiretroviral medications, and the risk of withdrawal and rebound phenomena, have prompted increasing interest in non-pharmacological treatments (131, 156). Non-pharmacological approaches, which include psychotherapy (e.g., cognitive-behavioral therapy) (157, 158), psychosocial support (e.g., meditation and yoga) (157–159), physical therapy (e.g., exercise) (160–162), and music therapy (163), have gained attention as complementary options. Clinical guidelines recommend psychotherapy and exercise therapy as first-line treatments, particularly for patients with mild to moderate depression (164). Studies indicate that exercise therapy offers significant benefits in addressing immune-mediated depression (IMD), particularly in patients with pronounced IMD features, and can serve as an alternative or adjunctive treatment to antidepressant medications (165). Moreover, a meta-analysis has demonstrated that resistance training provides clear antidepressant effects in patients without major physical comorbidities, highlighting its potential for clinical application in the comprehensive management of depression (166). These psychosocial intervention strategies are characterized by low technical requirements, high cost-effectiveness, and strong cultural adaptability, making them particularly suitable for implementation in resource-limited low- and middle-income countries. Overall, the increasing body of scientific evidence supports the integration of exercise therapy and psychotherapy as essential components of a comprehensive strategy for managing depression in PLWH.

### 4.5 Optimization of antiviral therapy regimens

Antiretroviral drugs used in the treatment of HIV infection include nucleoside reverse transcriptase inhibitors (NRTIs), NNRTIs, PIs, integrase strand transfer inhibitors (INSTIs), and fusion inhibitors. While these drugs effectively suppress HIV replication, lower viral load, enhance immune function, and significantly reduce the risk of drug resistance, certain medications, particularly NNRTIs, are associated with CNS toxicity, which may increase the risk of anxiety and depression (167, 168). These side effects can diminish treatment adherence, quality of life, and disease prognosis in PLWH, and may also lead to discontinuation of therapy. A prospective observational study involving 129 PLWH found that, on average, each participant experienced 2.4 neuropsychiatric disorders, with 89.9% of patients changing their treatment regimen due to symptoms such as sleep disturbances (75.2%), anxiety (65.1%), and depression (38.7%) induced by EFV (170). Another real-world study assessing discontinuation rates in patients on DTG-based regimens revealed that 13.7% of participants discontinued treatment due to intolerance, primarily due to neuropsychiatric symptoms such as sleep disturbances, anxiety, and depression (51).

Although CCR5 antagonists (e.g., Maraviroc), post-attachment inhibitors (e.g., Ibalizumab), and capsid inhibitors (e.g., Lenacapavir) hold significant therapeutic value in specific populations, their clinical use remains limited by targeted indication requirements and high treatment costs. Existing clinical trial data indicate that these three classes of drugs have not yet reported clinically significant neuropsychiatric side effects (169–171). However, their long-term neuropsychiatric safety in the real-world setting, particularly in individuals with comorbid mental health disorders, still requires further research for validation.

Maintaining good adherence to ART is a key factor in preventing viral rebound and associated neuroinflammation. It is important to note that depressive symptoms themselves may reduce medication adherence, highlighting the need for clinicians to closely monitor and intervene promptly when depressive symptoms occur in PLWH. For patients experiencing neuropsychiatric side effects, prompt adjustment of the treatment regimen is crucial to prevent further exacerbation of psychiatric symptoms. Studies have shown that compared to EFV(a NNRTI)-containing regimens, DTG (an INSTI)-based regimens, an integrase strand transfer inhibitor (INSTI), exhibit superior safety with a lower incidence of ADRs (172). According to the HIV Antiretroviral Therapy Guidelines for Adults and Adolescents, published by the U.S. Department of Health and Human Services (DHHS) on September 12, 2024, INSTIs such as DTG or bictegravir are recommended for ART due to their excellent virological suppression and favorable tolerability (173). Additionally, since the mechanism of action of NRTIs does not involve the CNS, they are particularly effective in minimizing neuropsychiatric symptoms. NRTIs are often combined with INSTIs, offering a lower risk of drug discontinuation due to toxicity and providing good overall treatment tolerability (174). In contrast, NNRTIs like EFV, which carry a higher risk of ADRs, should be used with caution in patients with existing neuropsychiatric symptoms. Overall, the combination of INSTIs and NRTIs is recommended to reduce the risk of psychiatric disorders, especially for patients with poor tolerance to other classes of drugs such as NNRTIs or PIs. It is particularly important to note that for PLWH presenting with unexplained cognitive impairment or neurological symptoms, even if plasma viral load is controlled, CSF HIV RNA quantification and drug resistance genotype testing should be considered to rule out the possibility of CSF viral escape (175).

## 5 Conclusion

PLWH face an increased risk for depression, anxiety, and other comorbid psychiatric disorders. However, the exact mechanisms underlying HIV-associated depression remain inadequately understood. This study integrates existing evidence linking HIV, depression, and chronic neuroinflammation, positing that the pathophysiological mechanisms involve intricate interactions across biological, psychological, and sociological dimensions. Biologically, factors such as neuroimmune activation, neurotransmitter dysregulation, HPA axis dysfunction, and structural and functional alterations in the brain are pivotal. Psychologically, the burden of chronic illness—encompassing stigma, social isolation, and trauma—plays a substantial role in the onset of depression. Sociologically, insufficient social support, economic stress, and a reduced quality of life further exacerbate the risk. These findings offer a novel perspective on the pathophysiology of HIV-associated depression, shedding light on its multifactorial nature. A particularly noteworthy aspect is the kynurenine pathway linking inflammation and neurotransmitter dysregulation: inflammatory signals alter the tryptophan metabolism process, which, on one hand, leads to serotonin depletion and, on the other hand, generates neurotoxic metabolites, thereby causing dysfunction in the glutamate system. Future research could focus on comparing the molecular characteristics (such as cerebrospinal fluid inflammatory marker profiles) of HIV-associated depression and primary depression, which would help further clarify the HIV-specific pathological mechanisms, particularly in terms of inflammation and neurotransmitter dysregulation related to the kynurenine pathway.

Addressing these risk factors necessitates comprehensive, targeted interventions. At the biological level, maintaining good adherence to ART is crucial for preventing viral rebound and associated neuroimmune activation. Depressive symptoms may reduce medication adherence, thus clinicians should closely monitor and intervene promptly. Optimizing ART regimens should involve prioritizing INSTIs with higher CNS safety, such as DTG, while avoiding the use of NNRTIs with significant neurotoxicity, such as EFV. Regular monitoring for neuropsychiatric adverse effects is also essential. In addition, neurotransmitter-modulating drugs (e.g., SSRIs/SNRIs), anti-inflammatory treatments, and HPA axis regulators may be used in combination, but potential interactions between these drugs and ART must be carefully evaluated. Psychologically, interventions like psychological support, stress management, and trauma-focused therapies are effective in improving mental health. Additionally, physical exercise and health education have potential in reducing depression risk. Sociologically, enhancing social support networks, mitigating economic stress, and improving patients’ quality of life can further improve therapeutic outcomes and promote adherence to treatment.

In conclusion, the prevention and treatment of HIV-associated depression require the establishment of a three-tiered intervention system: (1) optimization of ART regimens; (2) provision of structured psychological support; and (3) enhancement of social care networks. This comprehensive strategy, encompassing pharmacological treatment, psychological support, and social dimensions, is key to reducing the risk of depression and improving overall health outcomes for patients. Future research should further explore the long-term effects and underlying mechanisms of these interventions, particularly in optimizing strategies for PLWH. Such research will establish the scientific foundation for more precise and effective intervention approaches.
